# Structural Characterization of the Enzymes Composing the Arginine Deiminase Pathway in *Mycoplasma penetrans*

**DOI:** 10.1371/journal.pone.0047886

**Published:** 2012-10-17

**Authors:** Pablo Gallego, Raquel Planell, Jordi Benach, Enrique Querol, Josep A. Perez-Pons, David Reverter

**Affiliations:** 1 Institut de Biotecnologia i de Biomedicina and Departament de Bioquímica i de Biologia Molecular, Universitat Autònoma de Barcelona, Barcelona, Spain; 2 Experiments Division, ALBA Synchrotron Light Source, Barcelona, Spain; National Institute for Medical Research, Medical Research Council London, United Kingdom

## Abstract

The metabolism of arginine towards ATP synthesis has been considered a major source of energy for microorganisms such as *Mycoplasma penetrans* in anaerobic conditions. Additionally, this pathway has also been implicated in pathogenic and virulence mechanism of certain microorganisms, i.e. protection from acidic stress during infection. In this work we present the crystal structures of the three enzymes composing the gene cluster of the arginine deiminase pathway from *M. penetrans*: arginine deiminase (ADI), ornithine carbamoyltransferase (OTC) and carbamate kinase (CK). The arginine deiminase (ADI) structure has been refined to 2.3 Å resolution in its apo-form, displaying an “open” conformation of the active site of the enzyme in comparison to previous complex structures with substrate intermediates. The active site pocket of ADI is empty, with some of the catalytic and binding residues far from their active positions, suggesting major conformational changes upon substrate binding. Ornithine carbamoyltransferase (OTC) has been refined in two crystal forms at 2.5 Å and 2.6 Å resolution, respectively, both displaying an identical dodecameric structure with a 23-point symmetry. The dodecameric structure of OTC represents the highest level of organization in this protein family and in *M.penetrans* it is constituted by a novel interface between the four catalytic homotrimers. Carbamate kinase (CK) has been refined to 2.5 Å resolution and its structure is characterized by the presence of two ion sulfates in the active site, one in the carbamoyl phosphate binding site and the other in the β-phosphate ADP binding pocket of the enzyme. The CK structure also shows variations in some of the elements that regulate the catalytic activity of the enzyme. The relatively low number of metabolic pathways and the relevance in human pathogenesis of *Mycoplasma penetrans* places the arginine deiminase pathway enzymes as potential targets to design specific inhibitors against this human parasite.

## Introduction

Mycoplasmas belong to the class *Mollicutes*, a widely distributed group of microorganisms evolved from Gram-positive bacteria by a reduction in their genomes. The loss of genetic information related to metabolic activities and biosynthetic capabilities for essential compounds has emphasized the parasitic mode (of life) of all known *Mycoplasma* species, with each specie usually showing a strict host and tissue specificity [1]. *Mycoplasma penetrans* is a human parasite which persistently colonizes the respiratory and urogenital tracts invading and surviving within infected cells [2]. Lacking a complete tricarboxylic acid cycle and cytochromes, most mollicutes obtain ATP mainly through glycolysis and fermentative metabolism of carbohydrates. However, some *Mycoplasma* species are nonglycolytic or nonfermentative, and for them arginine catabolism has been considered, though not conclusively established, as the major source of energy [3]. In these cases, arginine degradation is done by means of the arginine deiminase (ADI) pathway, an anaerobic route found in Bacteria [4], Archaea [5], and amitochondrial Eukarya [6]–[8]. In mycoplasmas, the ADI pathway varies at the genomic level, ranging from the complete absence of all the genes in some species, to their presence, even though not necessarily functional, in others [9]. In this regard, *M. penetrans* possesses and expresses the complete set of the ADI pathway genes at the same time while exhibiting a fermentative metabolism.

The ADI pathway is composed by three catalytic enzymes: arginine deiminase (ADI, EC 3.5.3.6), ornithine carbamoyltransferase (OTC, EC 2.1.3.3) and carbamate kinase (CK, EC 2.7.2.2). This pathway performs the conversion of arginine to ornithine, ammonia, CO_2_ and ATP, yielding one mole of ATP per mole of arginine. ADI catalyzes the first step of the argininine degradation pathway, the deimination of arginine, producing citrulline and ammonium. Subsequently, OTC processes citrulline and uptakes inorganic phosphate to generate ornithine and carbamoyl phosphate. OTC enzymes can also be anabolic and catalyze the reverse reaction. The anabolic OTC can synthesize citrulline to be utilized in the arginine biosynthetic pathway or in the urea cycle. In this sense, some organisms, such as *Pseudomonas aeruginosa,* encode different OTCs genes dedicated to either the ADI pathway or to the arginine biosynthetic pathways [4], [10], [11]. In the last step of the ADI pathway, CK catalyzes the hydrolysis of the carbamoyl phosphate to CO_2_ and ammonia, while the phosphate group is used to phophorylate ADP to generate ATP. Similarly to OTCs, in some organisms such as *Pyrococcus furiosus,* CK can catalyze the opposite reaction synthesizing carbamoyl phosphate from carbamate and ATP [12].

The absence of the arginine deiminase gene (ADI) in the human genome, and the presence and importance of it in pathogenic protozoa like *Giarda intestinalis* [13], [14] and pathogenic bacteria like *Pseudomonas aeruginosa* [15]–[17] (primarily infecting immuno-compromised patients [18]), make the ADI enzyme an attractive therapeutic target for some parasitic and bacterial infections. Additionally, ADI might represent a potential tumor growth inhibitor [19]–[22]. ADI structures and reaction mechanisms have been described in detail in *Pseudomonas aeruginosa* [23], [24] and *Mycoplasma arginini* [25]. In the present work we have resolved the structure of ADI from *Mycoplasma penetrans* at 2.3 Å, and comparison to previous substrate-bound structures of ADI from *M.arginini* reveals substantial structural rearrangements in the active site of the enzyme upon binding of the substrate.

As previously mentioned, OTC (ornithine carbamoyltransferase) can be both anabolic or catabolic in some microorganisms [10], [26], possessing different genes for both catabolic and anabolic reactions, such as in *P. aeruginosa*. The anabolic reaction is related to the arginine biosynthesis in plants and to the urea cycle in the mitochondria in mammals [27], [28]. OTC is closely related to the aspartate transcarbamoylase (ATCase, EC 2.1.3.2) [29], a well-known example of an allosteric enzyme in the biosynthesis of pyrimidine. All transcarbamylases or carbamoyltransferases share the same basic overall protein fold which contains two domains: a CP-binding domain (carbamoyl phosphate binding domain) and an amino acid binding domain. The basic quaternary structure organization is a homotrimer, with the active site located at the interface between monomers [30], [31]. OTC proteins have a wide average range of quaternary structures, the most basic form being a homotrimer [32]. However, in some cases, such as *Lactobacillus hilgardii*, the quaternary assembly is an homohexamer, containing two homotrimers [33]. Additionally, a dodecameric assembly containing four homotrimers has been observed in the catabolic OTC of *P.aeruginosa* [31] and in the hyperthermophilic *Pyrococcus furiosus* [34]. Dodecameric assemblies of OTCs have been suggested to be involved in thermophilic resistance or in allosteric enzyme regulation by cooperativity [31], [35,35]. The two crystal structures of OTC from *Mycoplasma penetrans* described in this work possess a quaternary dodecameric assembly, which, despite a similar organization, is unique to *Mycoplasma penetrans* at the interface between the catalytic homotrimers and contain particular interactions.

Carbamate kinase (CK) generates ATP from carbamoyl phosphate and ADP, and this constitutes the last step in the fermentative catabolism of arginine, agmatine and allantoin/oxalurate [4], [36]–[40]. The absence of this protein in animals and its importance in generating energy in some pathogenic microorganisms like *Giardia intestinalis* or *Trichomonas vaginalis* makes CK an attractive therapeutic target [7], [41]. Structures of CK bound to ADP and to sulfate ions have already been described in two different organisms, *Enterococcus faecalis* and *Pyrococcus furiosus* [42]–[44], and in both cases there was a substantial structural rearrangement upon binding of substrates. The structure here of CK from *M.penetrans* differs from the previous structures by the presence of two sulfate ions, one in the carbamoyl phosphate pocket and another in the β-phosphate ADP binding pocket. Particularly interesting in the *M.penetrans* CK structure described here, is the “open” and non-catalytic conformation of the PSD subdomain, despite the presence of a sulfate ion in the carbamoyl phosphate-binding pocket.

## Results

### Protein expression and purification

The expression of arginine deiminase (ADI) from the pET-19b recombinant plasmid as described in Methods produced mostly insoluble protein. Transcription analysis of the *M. penetrans arc* operon, performed by primer extension, indicates the existence of two transcription start sites (unpublished results), giving rise upon translation, to the above described long-ADI and to a short-ADI form initiating at Met43 and ending at Lys452. Remarkably, pairwise and clustal alignments between ADI sequences from different *Mycoplasma* species and from *M. penetrans* start around Met43 residue of the short ADI-form, leaving N-terminal extensions (i.e., residues 1–42) without alignment in relation to the homologous sequences. To check if the recombinant expression of short-ADI would overcome the solubility problems encountered with long-ADI, the corresponding coding sequence was amplified from plasmid pBE-arcA by using adi-7 and adi-6 as forward and reverse primers, respectively (see Table S1). The 5′-end of adi-7 primer also bears an NdeI restriction site, allowing the cloning of the amplified short-ADI coding sequence into the pET-19b expression vector, as described for the long-ADI form. The same protein purification protocol described in Methods was applied to the short-ADI form. The recombinant production of short-ADI substantially improved from the production of the full length or long-ADI form in terms of both expression yields and solubility. Gel filtration of short-ADI, as expressed in *E.coli,* indicated the formation of a dimer, probably representing the biological unit of the protein. The final protein yield was 4 mg of recombinant ADI per liter of culture.

Recombinant production of full-length *M. penetrans* OTC in *E.coli* yielded a high level of expression, and the protein was easily purified by Ni^2+^-affinity interaction through the N-terminal His-tag from the pET-19b vector. Gel filtration chromatography of recombinant OTC indicated the presence of high molecular oligomerization forms, probably corresponding to a dodecameric structure, although other lower molecular weight oligomers could also be isolated. The final protein yield was 15 mg of recombinant OTC per liter of culture. Full-length carbamate kinase (CK) was also produced in *E.coli* in high yields and subsequently purified by Ni^2+^-affinity chromatography. Recombinant *M. penetrans* CK also eluted from the gel filtration column mainly as a dimer, suggesting that such a form might also correspond to the biological unit of the enzyme. The final protein yield was 6 mg of recombinant CK per liter of culture.

Before attempting the crystallization studies, the functionality of the three purified enzymes was tested by performing specific activity assays [45]. Activity data of ADI, OTC, and CK measured from crude cell extracts of *M. penetrans*, compared to the enzymes expressed in *E. coli,* allowed us to conclude that the obtained recombinant proteins behave as their native counterparts (data not shown).

### Crystal structure of arginine deiminase (ADI) from Mycoplasma penetrans

The polypeptide chain of ADI can be clearly and completely traced in the electron density maps from Gly54 to Lys452 (Figure S1). Crystals of *M.penetrans* ADI display a large unit cell composed of twelve molecules in the asymmetric unit. All the molecules in the asymmetric unit display a similar structure, with an rms deviation of 0.25 Å for the mainchain. Based on results obtained by gel-filtration chromatography, the biological unit can be considered to be a dimer (making up 6 dimers in the asymmetric unit). Structural alignment software (PDBefold) shows homology with the only other two known ADI structures, from *Mycoplasma arginini* (PDB code 1LXY) and from *Pseudomonas aeruginosa* (PDB code 1RXX). These structures display rms deviations with *M.penetrans* ADI of 1.54 Å and 1.85 Å, for 391 and 360 aligned residues with a pair-wise sequence identity, of 59% and 28%, respectively.

The overall protein fold and the secondary structure elements found in previous ADI structures are conserved in *M.penetrans* and consist of the repetition of a three-stranded mixed β sheet and a helix forming five ββαβ modules in a cyclical arrangement (Figure S1). This 5-fold pseudosymmetric moiety is also characteristic of other arginine-catabolizing enzymes such as Arg:Gly amidinotransferase (AT, [46]; PDB code 1JDW), dimethylarginine dimethylaminohydrolase (DDAH, [47]; PDB code 1H70) and agmatine deiminase (AgDI, [48]; PDB code 2JER). This characteristic protein fold seems to have evolved to interact and catalyze reactions containing guanidinium substrates, such as arginine. In the case of the arginine deiminase enzymes (ADI), this 5-fold pseudosymmetric α/β modular domain also contains an additional 85-residue α-helical domain between the first and second module (helices α4 to α8, see Figure S1). The role of this five-helix bundle domain, that is absent in the AT, DDAH and AgDI structures, is still not clear, but it has been speculated that it can participate in the activation of processes such as apoptosis [25].

**Figure 1:**
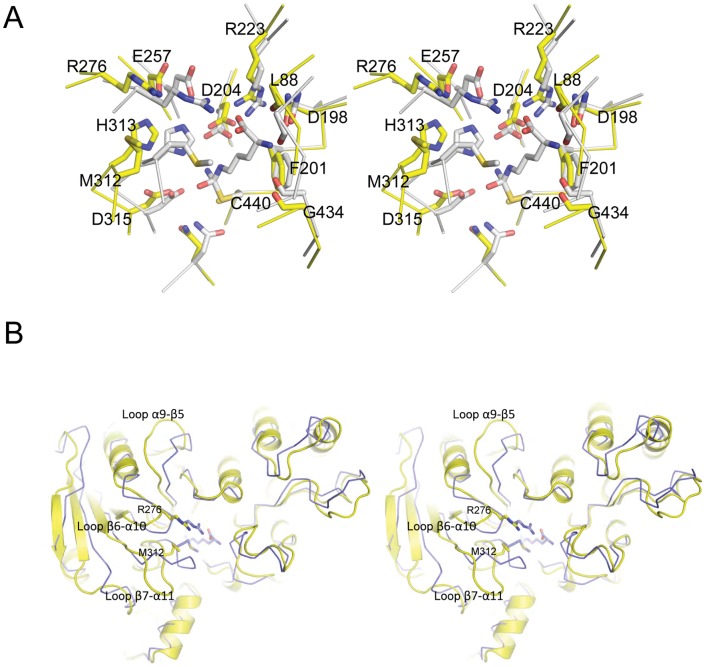
Conformational changes on the ADI structures upon substrate binding. (A) Stereo view representation of the superposition of the open active-site of *Mycoplasma penetrans* ADI (yellow) with closed active-site of the *Mycoplasma arginini* ADI in complex with arginine (white). ADI residues involved in the substrate binding and catalysis are labeled and shown in stick representation. (B) Stereo view ribbon representation of the superposition between the *Mycoplasma penetrans* ADI (yellow) with the *Mycoplasma arginini* ADI in complex with arginine (blue). Three major mobile loops of the ADI structure are labeled.

The active site of the enzyme is located approximately at the center of the structure, forming a buried and compact substrate-binding pocket. Previous structures of *M.arginini* ADI in a covalent complex with two L-arginine substrate intermediates ([25]; PDB code 1LXY and 1S9R) defined the binding pocket and the catalytic residues of the enzyme. In our apo-form structure of ADI from *M.penetrans*, all the substrate binding residues for the enzymatic reaction are conserved, but need to undergo substantial structural rearrangements to carry out the catalytic reaction. The apo-form ADI structure of *M.penetrans* would correspond to the “open” conformation of the enzyme, whereas the intermediate-substrate bound ADI structure of *M.arginini* would represent the “closed” conformation (Figure 1). As observed for many enzymes that follow an induced-fit mechanism, the occurrence of “open” and “closed” conformations are necessary to permit the correct catalytic reaction, which allows for the entrance and the release of the substrate and end product, respectively.

In the intermediate-bound ADI structure from *M.arginini* (PDB code 1LXY), the amino and carboxylate groups of the arginine substrate establish polar and charged interactions with Leu44, Asn155, Arg180, Arg232 and Gly392 (Leu88, Asn198, Arg223, Arg276 and Gly434 in the *M.penetrans* numbering). In our apo-form structure of *M.penetrans* ADI, the carbonyl groups of Leu88 and Gly434 are moved 2 Å away from their binding positions with the amino group of the arginine substrate, whereas Asn198 is located in a similar position. Arg223 and Arg276 (*M.penetrans* numbering), which bind the carboxylate group of the arginine substrate, undertake substantial movements of approximately 4 Å (particularly for Arg276) from their positions in the “open” to the “closed” conformations (Figure 1). The fixation of the aliphatic moiety of the arginine substrate is established by hydrophobic interactions with the Phe158 and Met268 sidechains (Phe201 and Met312 in the *M.penetrans* numbering). Comparison between the “closed” and our apo-form of *M.penetrans* ADI (“open” conformation) indicates that, whereas Phe201 does not move from its location, Met312 needs to move approximately 3 Å to interact with the substrate during the catalytic reaction.

The catalytic reaction of the ADI enzyme is performed by the catalytic triad composed of Cys440, His313 and Glu257, where the sulfur of Cys440 catalyzes a nucleophilic attack on the guanidino carbon of the arginine substrate. The role of Asp204 and Asp315 is also crucial for the stabilization of the tetrahedral intermediates during the catalytic reaction, as observed in the two intermediate complexes of ADI from *Mycoplasma arginini* and *Pseudomonas* [24], [25]. In our “open” conformation of *M.penetrans* ADI, Cys440 is located in a similar position as in the “closed” conformation, whereas His313 and Glu257 need to move about 3 Å to interact with the arginine substrate. Asp204 and Asp315 also move in this conformation, albeit to a lesser degree (Figure 1).

Therefore, it is essentially the movement of three loops containing some of the catalytic and binding residues of the active site pocket that is needed to switch from the “open” to the “closed” conformation of the enzyme, namely the loops α9-β5, β6-α10 and β7-α11 (Figure 1B). These three “mobile” loops are essentially shaping one side of the active site pocket, whereas the other side does not seem to undertake any substantial rearrangement upon substrate binding. A similar substrate induced-fit mechanism from the “open” to the “closed” conformation has also been previously described for the *Pseudomonas aeruginosa* ADI [23], [24], which has a 28% sequence identity with the ADI from *Mycoplasma*. Interestingly, a notable difference in the *P.aeruginosa* apo-form of ADI is the presence of an arginine residue, which corresponds to Met435 in *M.penetrans*, which is located in the middle of the active site pocket forming a salt bridge with an aspartate residue. This interaction needs to be disrupted and the arginine side chain displaced from the active site to allow the binding of the substrate. This does not occur in the *Mycoplasma* ADI and might be a reason for the large disparity in affinities for the substrate binding displayed by two orders of magnitude difference in the *K*_m_ constant [24].

### Crystal structure of ornithine carbamoyltransferase (OTC) from Mycoplasma penetrans

Two different crystal forms of the OTC from *M.penetrans* have been refined. The first one belonged to the P2_1_3 space group and was refined to 2.6 Å, and the second one belonged to a P321 space group and was refined to 2.5 Å. In both crystal forms the polypeptide chain of *M.penetrans* OTC can be clearly and completely traced in the electron density maps (from Met1 to Tyr342). The asymmetric unit of *M.penetrans* OTC is composed of four molecules in both crystals forms, displaying an average rms deviation of 0.35 Å between the four molecules. Structural alignment server (PDBefold) shows high homology with many OTC structures deposited in the PDB data bank, being the top ones from *Pseudomonas aeruginosa* (PDB code 1DXH) and from *Escherichia coli* (PDB code 1AKM). These structures display rms deviations compared with *M.penetrans* OTC of 1.26 Å and 1.21 Å, for 328 and 309 aligned residues with a sequence identity of 47% and 46%, respectively.

Despite the different space groups of the two crystal forms, in both cases an identical dodecameric quaternary structure was observed with an exact 23 point-group symmetry. In the two crystal forms this dodecameric structure was predicted by the PISA server (http://www.ebi.ac.uk/msd-srv/prot_int/pistart.htlm) and might correspond to the biological unit of the protein. The elution time in the gel filtration purification of the recombinant *M.penetrans* OTC also corresponds to the formation of a dodecameric quaternary structure. In most cases the oligomeric structure of OTC corresponds to a homotrimer, which represents the catalytic unit of the enzyme [32]. Only three OTCs have been reported to possess a similar dodecameric quaternary structure as *M.penetrans,* OTCs from *Pseudomonas aeruginosa* ([31]; PDB code 1ORT), *Pyrococus furiosus* ([34]; PDB code 1ALS) and *Thermotoga maritima* (PDB code 1VLV; only deposited). Remarkably, two of them are thermophiles, suggesting a potential role of the quaternary compact structures in high temperature protection in those environments. In all cases the dodecamer structure is formed by the interaction of four homotrimers disposed in a tetrahedral manner [34], [49], [50]. The four three-fold symmetry axes, located at the vertexes of the tetrahedral, are perpendicular to the center of the opposite homotrimer, and the three two-fold symmetry axes pass through the middle of opposite edges.

The trimeric structure of OTC is conserved in all known structures of OTCs and represents the catalytic unit of the enzyme, displaying one active site per monomer (Figure S2). As with all other known OTCs structures, the monomer consists of two domains of approximately similar size, the carbamoyl phosphate-binding domain (or polar domain, residues 1–150) and the ornithine-binding domain (or equatorial domain, residues 151–320). Each domain is named according to their respective involvement in substrate recognition. The secondary structure of each domain is made up of a five-stranded parallel β-sheet surrounded by α-helices, with helices α5 and α14 of *M.penetrans* OTC linking the two domains (Figure S2). The active site is located in a cleft formed between the two domains, and by comparison with other OTC structures, residues from both domains are involved in the catalytic reaction. By comparison with OTC structures bound to substrate analogs [51], [52], the relative positions of residues binding the carbamoyl phosphate moiety are mainly conserved in the *M.penetrans* OTC, including the positive pocket shaped by Ser59, Thr60, Arg61, Thr62, Arg110, His137, Gln140 and Arg328 (*M.penetrans* OTC numbering). Structural comparison between the apo- and the substrate-bound forms of OTCs [51], [52] indicate that the carbamoyl phophate-binding domain displays less flexibility than the ornithine-binding domain, in which larger structural rearrangements are induced during the catalytic reaction. Thus, whereas the catalytic patch formed by residues His275, Cys276 and Leu277 does not vary its position between the apo- and the substrate-bound forms in the ornithine-binding domain [51], [52], the loop between Trp237 and Phe247 displays a different conformation in the apo form of the *M.penetrans* OTC. Interestingly, this loop constitutes the main binding site for ornithine and is formed by the conserved DxxxSMG motif (between β8 and α10) in all OTC structures, except in our structure of *M.penetrans* OTC, in which methionine is substituted by leucine. The movement of this SMG-loop seems to be essential for binding of the second substrate and product release during the catalytic reaction [51].

The interfaces between monomers within the catalytic homotrimer are located mainly within the carbamoyl phosphate-binding domain, and are basically formed by the α2 helix and part of the last α14 helix that interacts with the region formed by the β2 strand and the α3 helix from the adjacent monomer (Figure S2). Analysis of the quaternary structure of the homotrimer structures of *M.penetrans* OTC (PISA server) displays a buried average interface of 590 Å^2^ between monomers. Each monomer contributes 60 residues to the interface, including 12 hydrogen bonds and 9 salt bridges. The total buried surface in the homotrimer structure is 1750 Å^2^ (indicating an energetically favorable formation of the homotrimer that constitutes the catalytic unit) with some of the interface residues as part of the active site.

**Figure 2:**
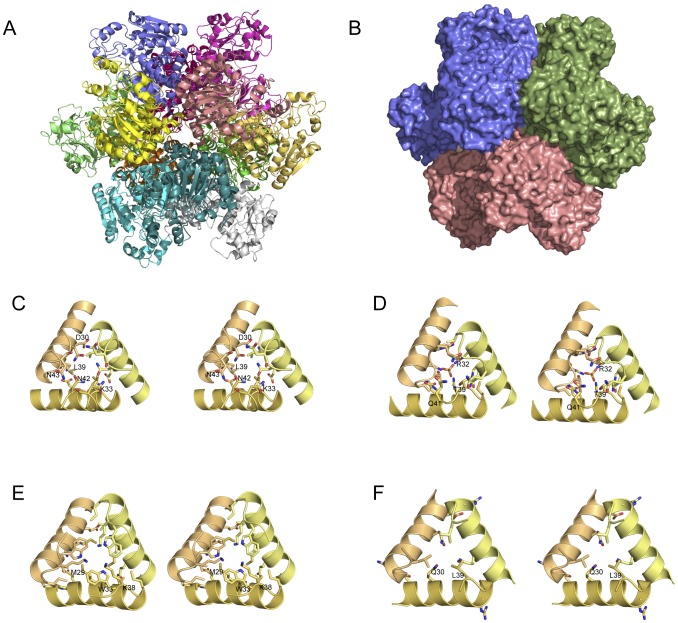
OTC dodecamer structure and the threefold interface between homotrimers. (A) Ribbon representation of the *Mycoplasma penetrans* OTC dodecamer. Each monomer is shown with a different colour. (B) Surface view of the *M. penetrans* OTC dodecamer. Each homotrimer is shown with a different color. (C, D, E, F) Stereo ribbon representation of the threefold interface between homotrimers. Residues involved in the dodecamer assembly are labeled and shown in stick representation for different species; (C) *Mycoplasma penetrans*, (D) *Pseudomonas aeruginosa*, (E) *Pyrococcus furiosus*, (F) *Thermotoga maritima*.

The interface between homotrimers that constitute the dodecameric structure is mainly located at the four 3-fold symmetry axes at the vertices of the tetrahedron (Figure 2). This interface is mainly formed by three α1 helices and the loop between α1 and β1 from three monomers from the different catalytic trimers located around each 3-fold symmetry axes. The interface between two monomers buries approximately 330 A^2^ that would represent a total of 990 A^2^ for each 3-fold symmetry axes interaction. Each monomer contributes 35 residues to the interface, mainly consisting of the aforementioned α1 helix residues, but with additional residues from the N- and C-terminal regions. Quaternary structure analysis by the PISA server indicates that the interface between two monomers contains 5 hydrogen bonds and 4 salt bridges. In Figure 2 the interface between homotrimers is located around the threefold symmetry axis at one top of the dodecamer. In the *M.penetrans* OTC dodecamer, this interface forms an intermolecular network of hydrogen bonds between Asn42 and Asn43 from the three monomers, which is unique to the sequence of *M.penetrans* OTC (Figure 2C).

Interestingly, the four known dodecameric structures of OTC (including the *M.penetrans* OTC) display completely different chemical profiles of the residues involved in the three-fold interface and only the relative positions of the main chain atoms can be superimposed. In the case of the *P.furiosus* OTC, the interaction around the threefold symmetry axis is mainly hydrophobic (Figure 2E), presenting major interactions between Met29, Trp33 and Lys38 [34]. In the case of the *P.aeruginosa* OTC, the threefold symmetry axis interface is basically charged and polar (Figure 2D), with major contribution of Arg28, Arg32, Thr39 and Gln41 [31]. The fourth dodecamer structure of an OTC is that of *Thermotoga maritima* (PDB code 1VLV; only deposited), and in this case the threefold symmetry axis interface presents a mixture of polar and hydrophobic interactions (Figure 2F). These different interfaces produce minor shifts and rotations between homotrimers in the global quaternary structure arrangement when a catalytic trimer is superimposed within the dodecamer.

In the case of *P.furiosus*, and perhaps in *T.maritima,* the domecameric quaternary structure has been involved in the extreme thermal stability of these microorganisms. It has been suggested that the thermophilic properties of the *P.furiosus* OTC are, in part, the result of the hydrophobic interfaces between homotrimers [49], [50]. In the case of *P.aeruginosa*, the dodecameric structure was first directly involved in the allosteric mechanism of the enzyme, which displays a marked cooperativity for carbamoyl phosphate [31], although it was later found than OTC homotrimers could also retain such cooperativity [53], [54]. In any case, the presence of higher oligomerization structures might be linked to an increase in the efficiency of the reaction in the ADI pathway, by means of thermal protection or by quaternary structure-induced cooperativity. Interestingly, a tunnel mechanism between OTC and the next catabolic enzyme of the ADI pathway, carbamate kinase (CK), has been suggested, as a means to protect from high temperatures and to efficiently transfer the labile (relatively unstable) product of the OTC reaction, carbamoyl phosphate [55].

### Crystal structure of carbamate kinase (CK) from Mycoplasma penetrans

The polypeptide chain of the *M.penetrans* CK can be traced in the electron density maps from Arg3 to Ser133 and from Lys157 to Ala309 (Figure S3). The gap in the electron density maps corresponds to the flexible PSD domain (“Protruding SubDomain”), and only residues at both ends of the PSD domain and the internal loop from Ala143 to Val150 could be partially modeled in the electron density, all displaying high temperature B-factors and giving as a result a poor agreement between R_work_ and the R_free_ in the final refined structure. Crystals of *M.penetrans* CK display a unit cell composed of one molecule in the asymmetric unit. Based on results obtained by gel-filtration chromatography, by predictions of the PISA server and by previous structures of CK from other organisms, the biological unit can be considered to be a dimer. Structural alignment server PDBefold shows strong homology with the other known CK structures, such as CK from *Pyrococcus furiosus* (PDB code 1E19), from *Giardia lamblia* (PDB code 2KZF) and from *Enterococcus faecalis* (PDB code 2WE5). These structures display rms deviations with *M.penetrans* CK of 1.23 Å, 1.59 Å and 1.54 Å, for 279, 266 and 271 aligned residues with sequence identities of 43%, 37% and 41%, for *P.furiosus*, *G.lamblia* and *E.faecalis*, respectively.

The overall protein fold and the secondary structure elements are conserved in *M.penetrans* CK and basically consist of a central eight-stranded parallel β-sheet, with six strands parallel in one orientation and two strands in an opposite orientation (β12 and β14), surrounded by α-helical elements at both sides of the central β-sheet (Figure S3). As previously described for the first CK structures from *E.faecalis* and *P.furiosus* [42], [43], the structure can be divided in two domains, with a large crevice in the middle that creates the binding sites for the two substrates, carbamoyl phosphate (CP) and ADP. The N-terminal domain (residues Met1 to Ala222), rich in α-helices, is composed by two sets of four stranded β-sheets, the first sheet constituted by four parallel strands from the central β-sheet and the second sheet smaller and formed by mixed β-strands. This domain contains three long α-helices, α1, α2 and α3, which make up the entire dimer interface, and the mobile PSD domain (“protruding subdomain”, from Lys126 to Val159), which is involved in the catalytic reaction by an overall “rigid body” movement upon substrate binding. The C-terminal domain (residues Asp223 to Ala309) is smaller and formed by the four mixed β-strands from the central β-sheet, surrounded by three α-helices. Whereas the binding residues for carbamoyl phosphate basically emanate from the N-terminal domain, the C-terminal domain is responsible for the binding of ADP. Interestingly, in our structure of *M.penetrans* CK, two sulfate ions from the crystallization buffer might be present in the β- and γ-phosphate-binding pockets of the synthesized ATP product molecule, with only present in such position in the CK structures from *E.faecalis* (PDB codes 2WE4 and 2WE5).

**Figure 3:**
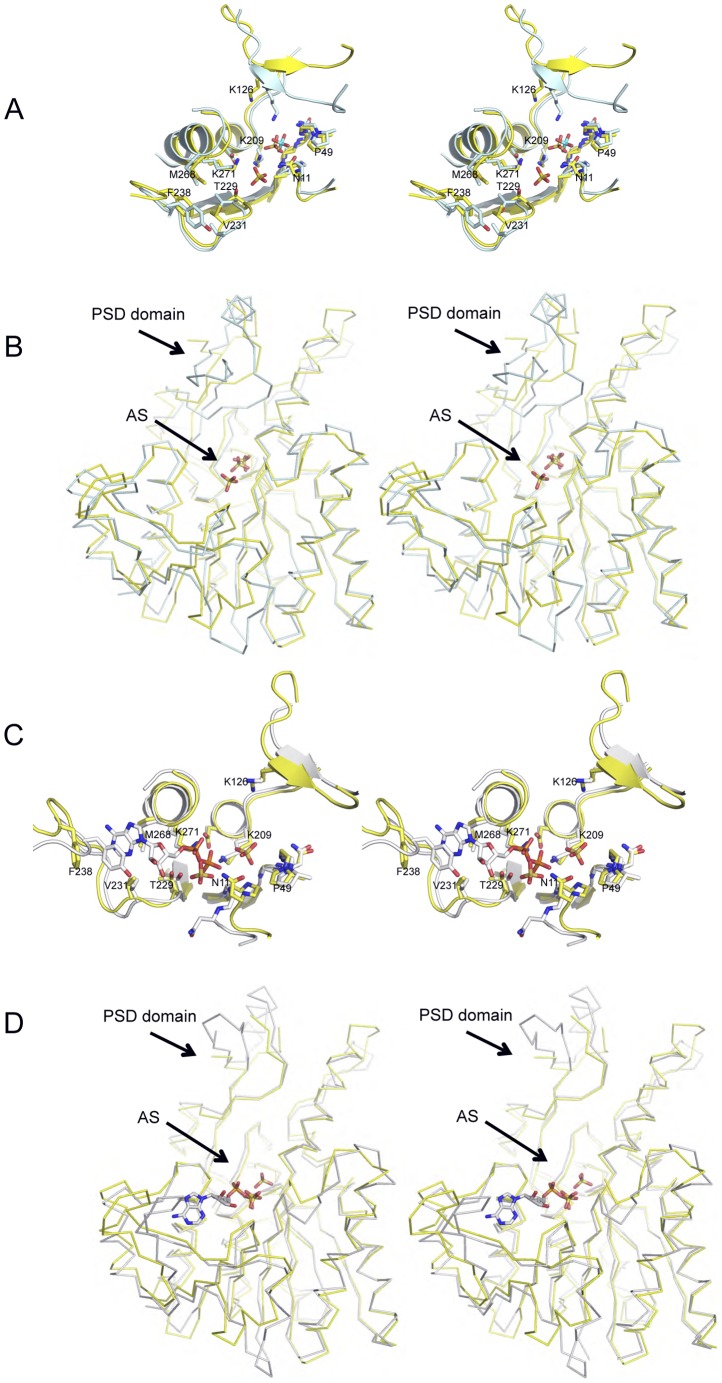
Conformational changes on the CK structures upon substrate binding. (A) Stereo view representation of the superposition of the active site of CK from *Mycoplasma penetrans* (yellow) and *Enterococcus faecalis* (ligth blue). Sulfate ions are shown in stick representation. CK residues involved in the substrate binding and catalysis are labeled and shown in stick representation. (B) Stereo view ribbon representation of the superposition of the CK from *Mycoplasma penetrans* (yellow) and *Enterococcus faecalis* (ligth blue). Sulfate ions are shown in stick representaion. (C) Stereo view representation of the superposition of the active site of CK from *Mycoplasma penetrans* (yellow) and from *Enterococcus faecalis* in complex with ADP (light blue). Sulfate ions and ADP molecule are shown in stick representation. CK residues involved in the substrate binding and catalysis are labeled and shown in stick representation. (D) Stereo view ribbon representation of the superposition of the CK from *Mycoplasma penetrans* (yellow) and *Enterococcus faecalis* (ligth blue) in complex with ADP.

In our current structure of *M.penetrans* CK, the location of some of the residues that have been suggested to contact the phosphate group of the carbamoyl phosphate substrate (CP) are conserved. These are the sidechain of Lys209 and the amino backbone nitrogens of Gly10, Asn11, Gly47, and Gly48. However, the putative coordination of the phosphate group of CP shown for the *E.faecalis* CK is not complete in *M.penetrans*, since the sidechain of Lys126 (Lys128 in the structure of the *E.faecalis* CK), which belongs to the mobile PSD domain, is not interacting with the sulfate ion. Interestingly, the inferred position of the PSD domain in the *M.penetrans* CK is in an “open” orientation, more similar to the *E.faecalis* CK-ADP bound structure (PDB code 2WE5) than to the *E.faecalis* CK-SO_4_-bound structure (PDB code 2WE4) (Figure 3). In our *M.penetrans* CK structure, the PSD domain can only be partially traced in the electron density maps, with all atoms displaying high values of temperature B-factors, indicating a strong tendency of this domain to shift between different conformations. The PSD domain has been postulated to act as a “rigid body” that is triggered upon the binding of the CP molecule to allow the catalytic reaction [43]. However, in our *M.penetrans* CK structure, despite the presence of a sulfate ion occupying a similar CP binding site as in the *E.faecalis* CK-SO_4_-bound (PDB code 2WE4), the PSD domain displays an “open” conformation, not productive for the phosphoryl transfer of the carbamoyl phosphate to the ADP molecule.

The second sulfate ion found in the *M.penetrans* CK structure is located approximately in a similar position as the β-phosphate of the ADP molecule observed in the *E.faecalis* and *P.furiosus* CK-ADP bound structures ([42], [43]; PDB codes 2WE5 and 1E19, respectively). Mimicking the ADP β-phosphate, the second sulfate ion interacts with the side chains of Lys271, Thr229 and the amino backbone nitrogen of Ala230. In our *M.penetrans* CK structure this sulfate ion is also at contact distance with the sidechain of Asn11 (3.24 Å), adopting the β1-α1 loop in a conformation similar to the CK-SO_4_-bound *E.faecalis* structure (PDB code 2WE5). Interestingly, the sidechain of Asn11 can potentially interact with both sulfate ions in our structure, the putative β-phosphate and mobile γ-phosphate of ATP, suggesting a participation of this residue in bridging the transfer of the phosphoryl group during the reaction of the enzyme. The other regions in the structure that might interact with the ADP molecule are in the “open” conformation, as observed in the CK-SO4-bound structure (PDB code 2WE5). These observations support the plasticity observed in most of the active-site regions of enzymes to allow catalysis and release of the end products.

In summary, the *M.penetrans* CK structure bound to two sulfate ions displays some differences in comparison to previous structures (CK-ADP from *E.faecalis* and *P.furiosus*; and CK-SO_4_ from *E.faecalis*). In the *M.penetrans* CK structure, despite the CP binding site being occupied by a sulfate ion, the PSD domain adopts an “open” orientation, which differs from the “closed” orientation observed in the CK-SO_4_ complex structure from *E.faecalis,* in which a sulfate ion occupies a similar position or in the *G.lamblia* CK structure ([56]; PDB code 3KZF), in which a glycerol molecule occupies the putative CP binding site. The orientation of the PSD domain in the *M.penetrans* CK resembles the PSD domains in the CK ADP-bound structures from *E.faecalis* and *P.furiosus*, but in both cases without any sulfate ions in the CP binding site (Figure 3D).

The movement of the PSD domain between an “open” and “closed” orientation to interact with the CP molecules has been described to be essential for the catalytic mechanism of the phosphoryl transfer between CP and ADP, allowing the interaction of the sidechain of Lys126 with CP molecule [43]. In our *M.penetrans* CK structure, the “open” orientation of the PSD domain with a sulfate ion occupying the CP binding site has not been observed, and might represent a structural snapshot of the binding of CP before the enzymatic reaction occurs. However/likewise, it could also be an example of the product complex before the release of the CP from the active site in the anabolic reaction of synthesis of the carbamoyl phosphate from carbamate [42]. Similarly, the binding of the second ion sulfate at the β-phosphate position of the ADP molecule indicates the strong binding tendency of this region to interact with charged ions (sulfates or phosphates). Remarkably, in a previous *E.faecalis* CK sulfate-bound structure, this second binding site pocket was not occupied by any sulfate ion.

## Conclusions

The arginine deiminase pathway (ADI) is the most widespread anaerobic route for arginine degradation in microorganisms and performs the conversion of arginine to ornithine, ammonia, and CO_2_, generating one mole of ATP per mole of arginine consumed. The ADI pathway constitutes an important source of energy in some microorganisms and like many other enzymatic pathways, it is also organized in a gene cluster to better regulate and optimize the production of the proteins. Here we have described the structure of the three enzymes that constitute the gene cluster in the ADI pathway in *Mycoplasma penetrans* (together with the specific membrane-associated arginine permease coded by *arc*D gene) and compared them to previous structures of the enzymes. Arginine deiminase (ADI), which catalyzes the first step in the ADI pathway, have been crystallized in its apo-form, and differs from the previous substrate-bound structures of *Mycoplasma arginini* by displaying an “open” conformation of the active site. The second enzyme of the pathway is ornitine transcarbamylase (OTC), which can catalyze the reaction in both directions and displays a dodecameric structure in the two crystal forms that have been crystallized. Dodecameric organization of OTC has been described in three microorganisms, *P.aeruginosa, P.furiosus* and *T.maritima,* in other organisms homotrimers can be found as the usual oligomeric form of this enzyme. Interestingly, the interfaces that form the OTC dodecamer in all these organisms are completely different, not showing any sequence homology between them and displaying either charged or hydrophobic interactions in the 3-fold symmetry axes of the dodecamer. Our current structure of *M.penetrans* OTC shows a novel interface forming the dodecamer. The last enzyme of the pathway is the carbamate kinase (CK), which is responsible for the formation of new molecules of ATP from carbamoyl phosphate. The crystal structure of CK contains two sulfate ions in the active site pocket, mimicking the binding of the β- and γ-phosphate of the ATP product molecule before the phosphorylation reaction has occurred. Interestingly, in our CK structure the position of the mobile PSD domain is in an “open” and non-catalytic conformation, despite the CP binding site being occupied by a sulfate ion, and might represent a structural snapshot of the binding of the substrates before the catalytic reaction occurs. The structural characterization of the enzymes of the ADI pathway in *Mycoplasma penetrans* has shed light to particular features of these proteins that eventually might provide new tools to interfere in the growing and proliferation of this pathogenic organism in humans.

## Methods

### Gene cloning and mutagenesis

A 3749-bp fragment (namely ArcABC) containing the genes for arginine deiminase (*arcA*), ornithine carbamoyltransferase (*arcB*), and carbamate kinase (*arcC*) was isolated from *Mycoplasma penetrans* GTU-54 strain (kindly provided by J. Baseman, University of Texas, San Antonio, USA) genomic DNA by PCR amplification using the set of primers Arc1penetr (forward) and Arc2penetr (reverse); see Table S1 for their respective sequences. The ArcABC fragment was blunt-end cloned into the *Eco*RV site of plasmid vector pBE, a derivative of pBSKII (Stratagene, CA) in which the multicloning site has been removed and substituted by a single *Eco*RV site [57]; the recombinant plasmid pBE-ArcABC was transformed into *E. coli* XL1-Blue strain. TGA is a stop codon in the universal genetic code but mycoplasmas translate such a codon as a tryptophan. Therefore, before attempting the heterologous expression in *E. coli* of mycoplasma gene products, any TGA in a coding region should be changed to TGG in order to incorporate the native Trp residue into the corresponding polypeptide. The *arcA* gene of *M. penetrans* contains two TGA codons that code for Trp317 and Trp339 in the arginine deiminase (ADI) amino acid sequence; equally, the *arcB* gene contains two TGA codons that code for Trp129 and Trp237 in the ornithine carbamoyltransferase (OTC). On the contrary, *arcC* gene coding for carbamate kinase (CK) does not contain any TGA codon. TGA codons were changed to TGG by site-directed mutagenesis, performing for each gene (*arcA* and *arcB*) six consecutive PCR steps, as outlined in Figure S4. The different couples of primers for each PCR reaction (numbered 1–6) and each gene (ADI and OTC) are indicated in Table S1, along with the length (in base pairs) of the amplified fragment. Mutagenic primers correspond to numbers 2, 3, 4, and 5; moreover, primers 2 and 4 are fully complementary to primers 3 and 5, respectively, and include the same point mutations (indicated with a bold character in Table S1). Phusion DNA polymerase (New England Biolabs) and 2 mM MgCl_2_ were used in all PCR reactions. Briefly, using the pBE-ArcABC recombinant plasmid as a template, a first PCR (PCR1 in Figure S4) amplifies from the start codon of the corresponding coding sequence to the first TGA codon to be mutated; a second PCR (PCR2) amplifies from the first TGA codon to the second one; and, finally a third PCR3 amplifies from the second TGA to the stop codon of the coding sequence. Next, using primers 1 and 4 and as DNA template the products of PCR1 and PCR2 reactions, PCR4 allowed to amplify from the start codon to the second TGA (already changed to TGG); in the same way, with the use of primers 3 and 6 and PCR2 plus PCR3 products as templates, PCR5 amplifies from the first TGA (TGG) to the stop codon. Note that PCR1, PCR2, and PCR3 products overlap at the ends including the mutated TGA codons (see Figure S4). Finally, the complete gene incorporating both changed TGG codons was obtained with PCR6 by using primers 1 and 6 and PCR4+PCR5 reaction products as templates; in this case, the complementary overlapping sequence between both PCR products corresponds to the DNA region expanding from the first to the second TGG. Not being necessary to change any TGA codon, the *arcC* gene was directly amplified from pBE-ArcABC using the corresponding primers 1 and 6 (ck-1 and ck-6 in Table S1, respectively). The products of the three PCR6 reactions (corresponding to *arcC*, and the mutated *arcA* and *arcB* genes) were cloned into the *Eco*RV site of the pBE vector and the recombinant plasmids pBE-ArcA, pBE-ArcB, and pBE-ArcC were transformed into *E. coli* XL1-Blue strain. For each gene, three independent clones were sequenced. Sequencing reactions with fluorescent dideoxynucleotides were performed using the Big Dye 3.0 Terminator kit (Applied Biosystems) following the manufacturer's instructions and analyzed in an ABI 3100 Genetic Analyzer (Applied Biosystems). The substitution of TGA by TGG codons was confirmed in the *arcA* and *arcB* genes, and no other change with regard to the native sequences (including the *arcC* gene) was observed.

### Protein expression and purification

For the recombinant expression of individual proteins, TGA mutated *arcA* (long-ADI, see below), *arcB*, and *arcC* genes were cloned into pET-19b plasmid vector (Novagen) as follows. Mutated genes were excised from the corresponding pBE derivative (see above) by digestion with *Nde*I and *Bam*HI restriction enzymes, whose recognition sequences were borne by the 5′-end of PCR primers 1 and 6, respectively (underlined in Table S1). The *Nde*I-*Bam*HI fragments were ligated to pET-19b expression vector, previously linearized with the same restriction enzymes, allowing the fusion of a His_10_-tag coding sequence to the 5′-end of each gene. Finally, the obtained recombinant plasmids were transformed into *E. coli* host expression strain BL21 (DE3).

Cells were grown at 37°C in Luria–Bertani (LB) growth medium supplemented with ampicillin (50 mg/L) to an optical density at 600 nm of 0.9 for ADI expression or 0.7 for OTC and CK expression. Protein expression was then induced by addition of 1 mM isopropyl β-D-1-thiogalactopyranoside (IPTG) to the culture medium, followed by shaking at 37°C for 3 hours. Induced cells were harvested by centrifugation, and resuspended in 5 mM imidazole, 0.5 M NaCl, 20 mM Tris-HCl (pH 7.9) (namely Binding Buffer), containing 1 mM phenylmethanesulfonyl fluoride (PMSF). Cells were disrupted by sonication, and the insoluble material removed by centrifugation at 40000×*g* for 20 minutes; supernatants were recovered and passed through a 0.45 µm filter. The histidine-tagged recombinant proteins were purified by loading the supernatants onto Hi-Trap Chelating Sepharose (GE Healthcare) chromatography columns equilibrated in Binding Buffer. Columns were washed with 60 mM imidazole, 0.5 M NaCl, 20 mM Tris-HCl (pH 7.9), and His-tagged retained proteins were eluted with 1 M imidazole, 0.5 M NaCl, 20 mM Tris-HCl (pH 7.9). Fractions containing the eluted proteins were pooled, dialyzed against 50 mM NaCl, 20 mM Tris–HCl (pH 8.0) buffer, and then loaded onto a HiLoad Superdex 200 26/60 gel-filtration column (GE Healthcare) previously equilibrated with the same buffer, as a final polishing purification step. Eluted fractions were analyzed by SDS-PAGE, and the purest samples were pooled, frozen in liquid-N_2_, and stored at −80°C. Protein concentration was estimated by the Coomassie-binding method of Bradford, using bovine serum albumin as a standard.

### Crystallization and data collection

Crystals of arginine deininase (ADI) were obtained at 18°C by sitting drop vapor diffusion methods. The reservoir solution contained 0.9M Trisodium citrate, 0.1M Sodium cacodylate pH 6.5. Single crystals appeared after 2 days from equal volumes of protein solution (5 mg/ml in 5 mM Tris pH 8.0, 50 mM NaCl) and reservoir solution. Crystals of ornithine carbamoyltransferase (OTC) were grown at 18°C by sitting drop vapor diffusion methods in two different reservoir conditions: in 0.2M ammonium acetate, 39% MPD, 0.1M tri-sodium citrate pH 5.5 (trigonal space group) and in 15% PEG20000 and 0.1M MES pH 6.5 (cubic space group). Crystals of carbamate kinase (CK) were obtained at 18°C by sitting drop vapor diffusion methods in a reservoir condition with 1.8M ammonium sulfate, 2% PEG400, 0.1M HEPES pH 7.0. All crystals were cryo-protected in reservoir buffer containing 12% glycerol and flash-frozen in liquid nitrogen prior to diffraction analysis. Diffraction data were recorded from cryo-cooled crystals (100K) at Grenoble beamline ID23-2. Data were integrated and merged using XDS [58] and scaled, reduced and further analyzed using CCP4 [59] (Table 1).

**Table 1:** Data collection and refinement statistics.

Data Collection	ADI	OTC (crystal 1)	OTC (crystal 2)	CK
Space group	P2_1_	P321	P2_1_3	P3_2_21
Cell dimensions				
a, b, c (Å)	120.5, 128.8, 220.3	183.6, 183.6, 117.3	167.9, 167.9, 167.9	51.8, 51.8, 174.2
α, β, γ (°)	90, 91.4, 90	90, 90, 120	90, 90, 120	90, 90, 120
Resolution (Å)^a^	30–2.3 (2.40– 2.30)	49–2.5 (2.64–2.50)	50–2.6 (2.74–2.60)	45–2.38 (2.51–2.38)
R_merge_ b	0.060 (0.367)	0.090 (0.436)	0.094 (0.367)	0.068 (0.388)
I/σ_I_	9.8 (2.0)	12.1 (2.6)	8.7 (2.4)	11.8 (2.5)
Completeness (%)	97.6 (96.1)	98.2 (89.7)	98.6 (95.4)	97.7 (92.2)
Redundancy	1.9 (1.9)	4.8 (3.4)	2.4 (2.3)	4.8 (4.5)
Refinement				
Resolution (Å)	30–2.3	49–2.5	48–2.6	45–2.5
No. Reflections	288889	74328	47453	9819
R_work_/R_free_ c	21.60/24.23	17.01/21.51	17.9/23.9	23.19/28.68
No. Atoms	39631	11190	11035	2558
No. aa protein	4788	1376	1368	309
Water	1615	418	339	33
R.m.s deviations				
Bond lengths (Å)	0.007	0.008	0.009	0.008
Bond angles (°)	1.33	1.154	1.206	1.155
PDB code	4E4J	4AMU	4ANF	4AXS

a Statistic for highest resolution shell is shown in parentheses.

b R_merge_  =  ∑ |I_i_ − < I >|/∑ I_i_ , where I_i_ is the ith measurement of the intensity of an individual reflection or its symmetry-equivalent reflections and <I> is the average intensity of that reflection and its symmetry-equivalent reflections.

c R_work_  =  ∑ ||F_o_| − |F_c_||/∑ |F_o_| for all reflections and R_free_  =  ∑ ||F_o_| − |F_c_||/∑ |F_o_|, calculated based on the 5% of data excluded from refinement.

### Structure determination and refinement

The structure of ADI was determined from the x-ray data by molecular replacement using the PDB from *Mycoplasma arginini* (PDB code 1S9S) as a model. The two structures of OTC were determined from the x-ray data by molecular replacement using the PDB from *Pseudomonas aeruginosa* (PDB code 1DXH) as a model. The structure of CK was determined from the x-ray data by molecular replacement using the PDB from *Enterococcus faecalis* (PDB code 2WE5) as a model. The initial electron density maps produced from molecular replacement programs were manually improved to build up complete models for ADI, OTC and CK using the program COOT [60]. Model refinement was performed with CNS [61] and Phenix [62]. ADI contained 12 molecules in the asymmetric unit and the Ramachandran analysis shows 95.36% of residues (4543) are in preferred regions, 4.47% of residues (213) are in allowed regions and 0.17% of residues (8) are in outlier regions. OTC crystal 1 contained four molecules in the asymmetric unit and the Ramachandran analysis shows 94.71% of residues (1288) are in preferred regions, 4.41% of residues (60) are in allowed regions and 0.88% of residues (12) are in outlier regions. OTC crystal 2 also contained four molecules in the asymmetric unit and the Ramachandran analysis shows 94.34% of residues (1283) are in preferred regions, 4.34% of residues (59) are in allowed regions and 1.32% of residues (18) are in outlier regions. CK contained one molecule in the asymmetric unit and the Ramachandran analysis shows 90.88% of residues (259) are in preferred regions, 7.37% of residues (21) are in allowed regions and 1.75% of residues (5) are in outlier regions. Refinement and data statistics are provided in Table 1. Structural representations were prepared with PyMOL [63].

### Accession codes

Protein Data Bank: Coordinates and structure factors from the three structures were deposited in the PDB data with accession ID codes 4E4J (MpADI), 4AMU (MpOTC), 4ANF (MpOTC) and 4AXS (MpCK).

## Supporting Information

Figure S1 **ADI structure and sequence alignment.** (A) Ribbon representation of the ADI structure from *Mycoplasma penetrans*. The α-helix and β- strands are represented in red and golden, respectively, and are labeled sequentially from N- terminus. (B) Surface representation of ADI from *M.penetrans*. AS indicates the active site pocket. (C) Surface representation of ADI from *M.arginini* (PDB code) in complex with arginine in the active site (colored in magenta). AS indicates the active site pocket. (D) Topology-based sequence alignment of ADI from *Mycomplasma penetrans*, *Mycoplasma arginini* and *Pseudomonas aeruginosa*. The secondary structural elements are labelled and shown above the sequence as rectangles or arrows for α-helix and β-strands, respectively. Active site residues are colored in red. Identical or highly conserved residues are highlighted in yellow.
(PDF)

Figure S2 **OTC structure and sequence alignment.** (A) Ribbon representation of the structure of the OTC monomer from *Mycoplasma penetrans*. The α-helix and β-strands are represented in red and golden, respectively, and are labeled sequentially from the N-terminus. Residues involved in the active-site are shown in stick representation. (B) Ribbon representation of the OTC homotrimer structure. (C) Topology-based sequence alignment of OTC from *Mycoplasma penetrans*, *Pseudomonas aeruginosa*, *Themortoga maritima*, *Pyrococcus furiosus*, *Lactobacillus hilgardii*, *Gleobacter violacius* and *Escherichia coli*. The secondary structural elements are labeled and shown above the sequence as rectangles or arrows for α-helices or for β- strands, respectively. Active site residues are colored in red. Identical or highly conserved residues are highlighted in yellow.
(PDF)

Figure S3 **Structure of the CK from M. penetrans and sequence alignment.** (A) Ribbon representation of CK from *Mycoplasma penetrans*. The α-helix and β-strands are represented in red and golden, respectively, and are labeled sequentially from the N-terminus. Residues involved in the active-site are shown in stick representation. (B) Ribbon representation of the dimer of CK. In one of the monomers, the N-terminal domain is in colored in light blue, the C-terminal domain in forest green and the PSD domain in lime green. The two sulfate ions are shown in stick representation. (D) Topology-based sequence alignment of CK from *Mycoplasma penetrans*, *Enterococcus faecalis*, *Pyrococcus furiosus* and *Giardia lamblia*. The secondary structural elements are labeled and shown above the sequence as rectangles or arrows for α- helices or forβ-strands, respectively. Active site residues are colored in red. Identical or highly conserved residues are highlighted in yellow.
(PDF)

Figure S4 **Mutagenesis strategy.** Scheme illustrating the strategy followed to change the two TGA, coding for tryptophan in *M. penetrans*, by TGG codons before the heterologous expression of *arc*A (ADI) and *arc*B (OTC) genes in *E. coli*. Numbered arrows indicate the primers (see Table S1 for primer sequences and features). The successive six PCR amplifications carried out are also indicated. Cloning of *arc*C gene, which does not contain any TGA codon, was performed in a single reaction (PCR6) using primers 1 and 6.
(PDF)

Table S1 **Primers.** Underlining indicates the restriction sites introduced at the 5′ end of selected primers. A bold lowercase character indicates the changed base in mutagenic primers.
(PDF)
