# Supplementary figures and images for: Structural Characterization of the Enzymes Composing the Arginine Deiminase Pathway in Mycoplasma penetrans
Source: PLoS One. 2012 Oct 17;7(10):e47886. doi: 10.1371/journal.pone.0047886 (PMC3474736; doi:10.1371/journal.pone.0047886)

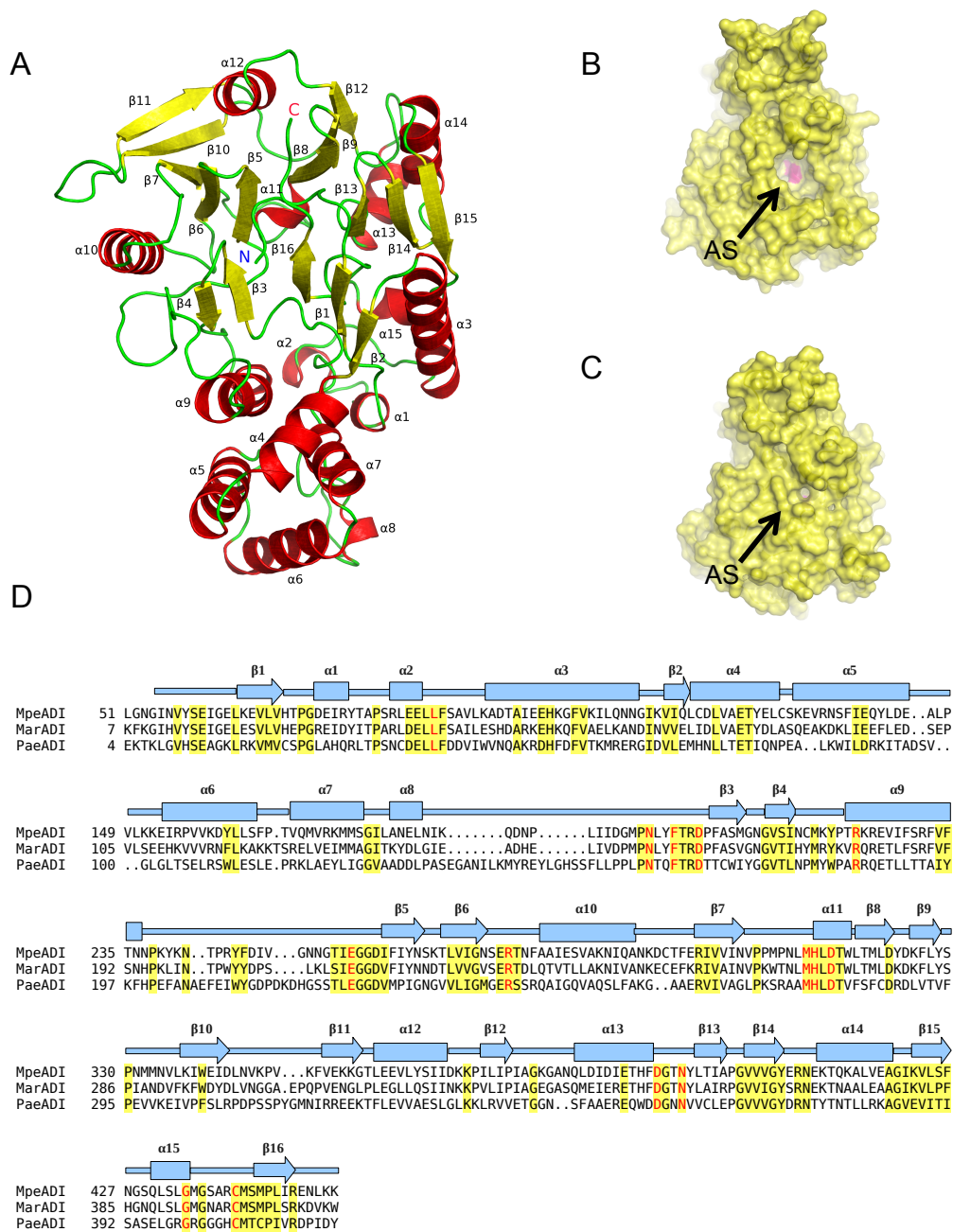

Figure S1

Supplement: Figure S1 — ADI structure and sequence alignment. (A) Ribbon representation of the ADI structure from Mycoplasma penetrans. The α-helix and β- strands are represented in red and golden, respectively, and are labeled sequentially from N- terminus. (B) Surface representation of ADI from M.penetrans. AS indicates the active site pocket. (C) Surface representation of ADI from M.arginini (PDB code) in complex with arginine in the active site (colored in magenta). AS indicates the active site pocket. (D) Topology-based sequence alignment of ADI from Mycomplasma penetrans, Mycoplasma arginini and Pseudomonas aeruginosa. The secondary structural elements are labelled and shown above the sequence as rectangles or arrows for α-helix and β-strands, respectively. Active site residues are colored in red. Identical or highly conserved residues are highlighted in yellow. (PDF) [file pone.0047886.s001.pdf]

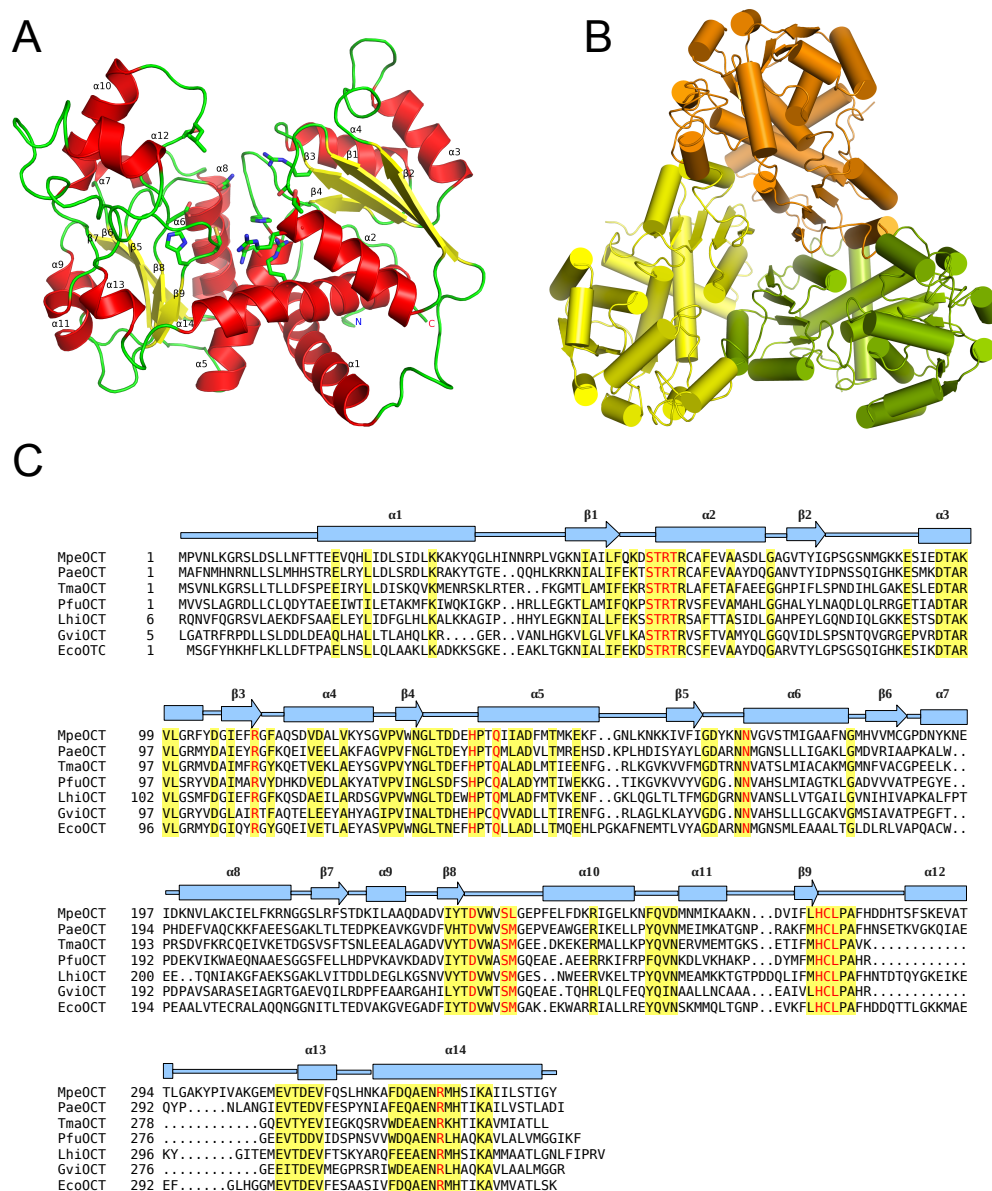

Figure S2

Supplement: Figure S2 — OTC structure and sequence alignment. (A) Ribbon representation of the structure of the OTC monomer from Mycoplasma penetrans. The α-helix and β-strands are represented in red and golden, respectively, and are labeled sequentially from the N-terminus. Residues involved in the active-site are shown in stick representation. (B) Ribbon representation of the OTC homotrimer structure. (C) Topology-based sequence alignment of OTC from Mycoplasma penetrans, Pseudomonas aeruginosa, Themortoga maritima, Pyrococcus furiosus, Lactobacillus hilgardii, Gleobacter violacius and Escherichia coli. The secondary structural elements are labeled and shown above the sequence as rectangles or arrows for α-helices or for β- strands, respectively. Active site residues are colored in red. Identical or highly conserved residues are highlighted in yellow. (PDF) [file pone.0047886.s002.pdf]

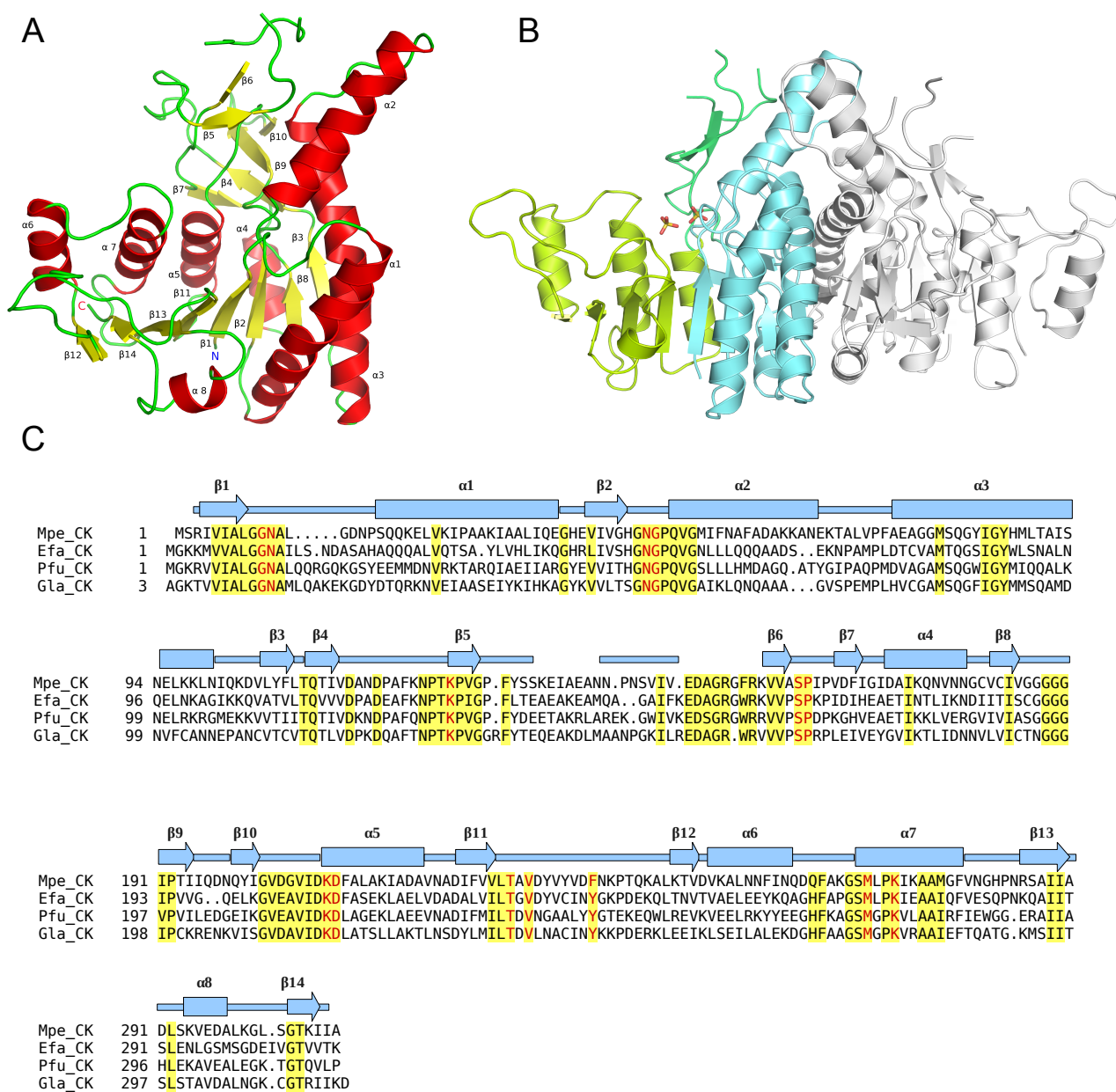

Figure S3

Supplement: Figure S3 — Structure of the CK from M. penetrans and sequence alignment. (A) Ribbon representation of CK from Mycoplasma penetrans. The α-helix and β-strands are represented in red and golden, respectively, and are labeled sequentially from the N-terminus. Residues involved in the active-site are shown in stick representation. (B) Ribbon representation of the dimer of CK. In one of the monomers, the N-terminal domain is in colored in light blue, the C-terminal domain in forest green and the PSD domain in lime green. The two sulfate ions are shown in stick representation. (D) Topology-based sequence alignment of CK from Mycoplasma penetrans, Enterococcus faecalis, Pyrococcus furiosus and Giardia lamblia. The secondary structural elements are labeled and shown above the sequence as rectangles or arrows for α- helices or forβ-strands, respectively. Active site residues are colored in red. Identical or highly conserved residues are highlighted in yellow. (PDF) [file pone.0047886.s003.pdf]

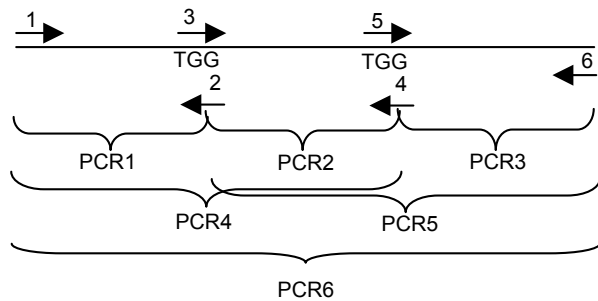

Figure S4

Supplement: Figure S4 — Mutagenesis strategy. Scheme illustrating the strategy followed to change the two TGA, coding for tryptophan in M. penetrans, by TGG codons before the heterologous expression of arcA (ADI) and arcB (OTC) genes in E. coli. Numbered arrows indicate the primers (see Table S1 for primer sequences and features). The successive six PCR amplifications carried out are also indicated. Cloning of arcC gene, which does not contain any TGA codon, was performed in a single reaction (PCR6) using primers 1 and 6. (PDF) [file pone.0047886.s004.pdf]
