# Supplementary material for: Structural Characterization of the Enzymes Composing the Arginine Deiminase Pathway in Mycoplasma penetrans
Source: PLoS One. 2012 Oct 17;7(10):e47886. doi: 10.1371/journal.pone.0047886 (PMC3474736; doi:10.1371/journal.pone.0047886)
Supplement: Table S1 — Primers. Underlining indicates the restriction sites introduced at the 5′ end of selected primers. A bold lowercase character indicates the changed base in mutagenic primers. (PDF) [file pone.0047886.s005.pdf]

Table 1.

Primer	Sequence (5'- 3')
Arc1‡	GTCGACCCACATGAGCTTAAAGGGATAG
Arc2‡	GTCGACTACTTGTTGGAGCTTGAGAAATTC
adi-1	CATATGTTGGTTATTACAATTGCACTAAATAT
adi-2*	CTAACATTGTTAA**c**CAAGTATCTAAATGCATTAG
adi-3*	CTAATGCATTTAGATACTTG**g**TTAACAATGTTAG
adi-4*	GGTTTAACATTTAGATCAATTTC**c**CAAATCTTTAATACATTC
adi-5*	GAATGTATTAAAGATTTG**g**GAAATTGATCTAAATGTTAAACC
adi-6	GGATCCTTATTTTTTTAAATTTTCTCTAATTAAAGG
adi-7	CATATGATGAGTAGTATTGATAAAAATTCAC
otc-1	CATATGATGCCAGTAAATTTAAAAGGAAGAAG
otc-2*	CTGTTAACCCATT**c**CAAACTGGAACTCCAG
otc-3*	CTGGAGTTCCAGTTTG**g**AATGGGTTAACAG
otc-4*	CTCCAAGAGATAC**c**CAAACGTCTGTATAG
otc-5*	CTATACAGACGTTTG**g**GTATCTCTTGGAG
otc-6	GGATCCCTAGTAACCAATTGTAGACAGAATG
ck-1	CATATGATGTCTAGAATTGTTATTGCTTTAG
ck-6	GGATCCTTAAGCAATTATTTTTGTACCACTTAATC

‡ Used to perform PCR amplification of the DNA fragment containing *arc*A, *arc*B, and *arc*C genes from *M. penetrans* genomic DNA.
* Mutagenic primers used to change the two TGA by TGG codons in each *arc*A and *arc*B coding sequence.
